# Evaluation of a New Shirt-Based Electrocardiogram Device for Cardiac Screening in Soccer Players: Comparative Study With Treadmill Ergospirometry

**DOI:** 10.14740/cr333w

**Published:** 2014-07-20

**Authors:** Oscar Fabregat-Andres, Adolfo Munoz-Macho, Guillermo Adell-Beltran, Xavier Ibanez-Catala, Agustin Macia, Lorenzo Facila

**Affiliations:** a Department of Cardiology, Consorcio Hospital General Universitario, Valencia, Spain; b Villarreal Club de Futbol SAD Medical Services, Villarreal, Spain; c Nuubo, Madrid, Spain

**Keywords:** Cardiac screening, Wireless device, ECG monitoring, Exercise test, Ergospirometry, Soccer

## Abstract

**Background:**

Prevention of cardiac events during competitive sports is fundamental. New technologies with remote monitoring systems integrated into clothing could facilitate the screening of heart disease. Our aim was to evaluate the feasibility of Nuubo system during a field stress test performed by soccer players, comparing results with treadmill ergospirometry as test reference.

**Methods:**

Nineteen male professional soccer players (19.2 ± 1.6 years) were studied. Wireless electrocardiographic monitoring during a Yo-Yo intermittent recovery test level 1 in soccer field and subsequent analysis of arrhythmias were firstly performed. Subsequently, in a period no longer than 4 weeks, each player underwent cardiopulmonary exercise testing in hospital.

**Results:**

During Yo-Yo test, electrocardiogram (ECG) signal was interpretable in 16 players (84.2%). In the other three players, ECG artifacts did not allow a proper analysis. Estimation of maximum oxygen consumption was comparable between two exercise tests (VO_2_ max 53.3 ± 2.4 vs. 53.7 ± 3.0 mL/kg/min for Yo-Yo test and ergometry respectively; intra-class correlation coefficient 0.84 (0.63 - 0.93), P < 0.001). No arrhythmias were detected in any player during both tests.

**Conclusions:**

The use of Nuubo’s technology allows an accurate single-lead electrocardiographic recording and estimation of reliable performance variables during exercise testing in field, and provides a new perspective to cardiac remote monitoring in collective sports.

## Introduction

Primary prevention of cardiac events in competitive sports has emerged as a key target of sports medicine. Screening protocol for cardiovascular abnormalities proposed by current guidelines [1, 2] consists at least of complete medical history, physical examination and resting 12-lead electrocardiogram (ECG). This screening strategy has provided solid evidence that may prevent sudden cardiac death in the athlete [3], although its large-scale implementation remains controversial [4].

In fact, due to the dynamic nature of ECG abnormalities in some channelopathies responsible for a high percentage of cases of sudden death in athletes, usefulness of resting ECG could be considered limited [5]. Thereby, some conditions such as increased body temperature, changes in autonomic status or increased heart rate are necessary for definitive diagnosis [6-8]. All these variables come together during intense physical exercise practice.

Ergospirometry as stress test is widely used in elite athletes. However, the accuracy of continuous and progressive tests to determine individual physical performance in team sports as soccer, which requires repeated anaerobic efforts with short resting times, remains still controverted, because the activity developed with treadmill test is suboptimally adjusted to the sport real practice.

For these reasons, new technologies with remote wireless monitoring systems integrated into clothing could facilitate the screening of heart disease in collective sports [9]. Secondly, these emergent technological tools could also provide a more accurate assessment of physical performance of the athlete as they allow the practice of specific exercise tests adapted to the sports field.

Nuubo’s dynamic ECG (nECG platform) is a new single-lead dynamic ECG wireless monitoring system that incorporates biomedical e-textile technology, and consists of three elements: an electronic device (nECG minder) attached to the garment that transmits the ECG signal via Bluetooth^®^ to a computer (among other signals like accelerometer); a biomedical shirt that captures the ECG signal via textile electrodes technology BlendFix^®^ (nECG shirt); and a software package that manages sessions, activities and users, and allows the visualization and analysis of data captured by the nECG minder (nECG suite) [10].

Our objective was to evaluate the applicability of Nuubo system during a stress test performed in field by professional soccer players, assessing both the ability of this device to screen cardiac arrhythmias during exercise and its capability to estimate individual physical performance using specific tests, compared with treadmill ergospirometry and test reference.

## Material and Methods

### Participants

Nineteen male professional soccer players from Villarreal B team of the Spanish second division B were studied. Although the team consists of 23 players, four were excluded because of injury. The study protocol was consistent with the institutional ethical requirements in accordance with the Declaration of Helsinki and all subjects were fully informed about the procedures to be used.

### Field test with Nuubo system

Yo-Yo intermittent recovery test (YYIRT) level 1 was performed at the beginning of training session after 10 min of warming. It was used as reference test to determine performance and indirect estimation of maximum oxygen consumption (VO_2_ max) in soccer players [11, 12]. An initial session was completed for 17 players. Other two injured players at the moment of first test, performed YYIRT 2 weeks later. All players wore an nECG shirt 15 min before exercise and the quality of the signal was checked by wireless transmission to a computer located in the field. Subsequently, ECG signal was analyzed using nECG suite software to detect arrhythmias and to determine parameters of heart rate variability (HRV). VO_2_ max was calculated using the formula: (distance in meters × 0.0084 + 36.4) [13].

### Ergospirometry

All players performed a treadmill exercise testing in hospital within a month after the YYIRT. A progressive, continuous, triangular, maximum protocol was used, consisting of warming at 5 km/h for 5 min, exercise at 8 km/h for 2 min with increases of 2 km/h each 2 min, and recuperation at 5 km/h for 5 min. Expired gases were analyzed in each ventilatory cycle and VO_2_ max was directly calculated.

### Other complementary tests

Before training, fasting blood test, 12-lead ECG at rest and blood pressure measurement were performed. Peripheral lactate was also analyzed before and immediately after the test using a Lactate Pro 2 Analyzer (Arkray Inc., Japan).

### Statistical analysis

Quantitative data were expressed as mean ± standard deviation and qualitative data as median and absolute range. Normal distribution was analyzed by Kolmogorov-Smirnov test. Comparisons between groups were made with *t*-test for continuous variables. Inter-methods agreement was evaluated by means of the intra-class correlation coefficient (ICC) and kappa index, and the relationship between both tests was also calculated using Pearson’s correlation coefficient (r). Differences were considered statistically significant when a P value < 0.05 was obtained. All analyses were performed using SPSS version 17.0 (SPPS Inc., USA).

## Results

Nineteen players completed both exercise tests. Baseline characteristics of the participants, blood pressure measurement, lactate values, YYIRT and ergospirometry results, and HRV parameters estimated by Nuubo system are shown in Table 1.

**Table 1:** Baseline Characteristics of Participants, Yo-Yo Test and Ergospirometry Results, and Heart Rate Variability Parameters Estimated by Nuubo System

General characteristics	n = 19
Age (years)	20.1 ± 1.8
BMI (kg/m^2^)	22.4 ± 1.5
Percentage body fat (%)	11.8 ± 2.0
Blood pressure (mm Hg)	113.3 ± 9.3/67.3 ± 7.2
YYIRT level 1	n = 19
Maximal heart rate (bpm)*	187.7 ± 3.8
Mean QTc (ms)*	398.9 ± 20.2
Distance (m)	2,006.3 ± 282.9
Median stage reached	18.7 (17.6 - 20.7)
Estimated VO_2_ (mL/kg/min)	53.3 ± 2.4
Basal lactate (mmol/L)	2.6 ± 0.7
Maximal lactate (mmol/L)	10.4 ± 1.7
Ergospirometry	n = 19
Maximal heart rate (bpm)	190.1 ± 5.5
Time (min)	16.7 ± 0.9
VO_2_ (mL/kg/min)	53.7 ± 3.0
Maximal lactate (mmol/L)	8.5 ± 3.3
Aerobic threshold (mL/kg/min)	28.5 ± 6.1
Anaerobic threshold (mL/kg/min)	38.5 ± 4.7
Heart rate variability parameters	n = 16
Mean RR interval (ms)	369.7 ± 27.6
SDNN (ms)	38.7 ± 19.0
rMSDD (ms)	19.1 ± 19.2
pNN50 (%)	2.82 ± 4.4
Mean triangular index	4.45 ± 1.5
LF/HF ratio	1.87 ± 1.0

BMI: body mass index; bpm: beats per minute; YYIRT: Yo-Yo intermittent recovery test; QTc: corrected QT interval; VO_2_: maximal oxygen uptake; g: unit of acceleration; SDNN: standard deviation of normal-to-normal RR intervals; rMSDD: square root of the mean squared differences of successive normal-to-normal RR intervals; pNN50: proportion of interval differences of successive normal-to-normal RR intervals; LF/HF ratio: ratio of low- to high-frequency power. *Calculated only on 16 adequate and interpretable ECG signals.

**Figure 1:**
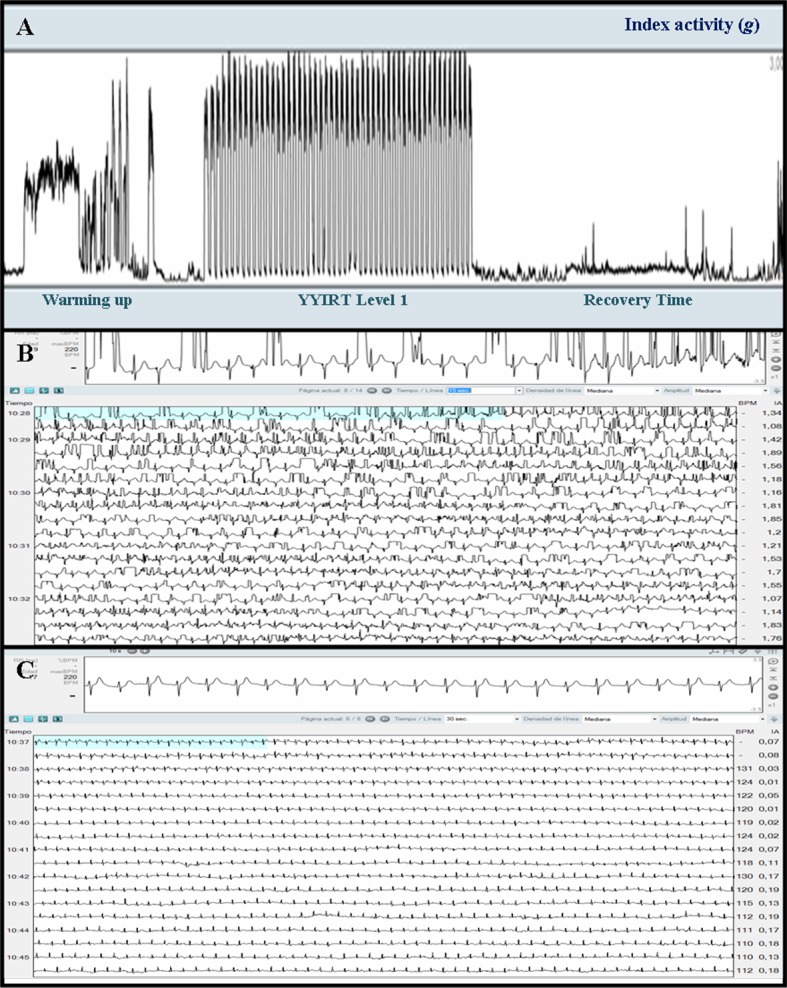
Wireless electrocardiographic monitoring recordings of soccer players during Yo-Yo intermittent recovery test level 1. (A) Activity index during the three phases of the test: warming up, Yo-Yo test and recovery. Physical activity expressed in units of acceleration (g). (B) ECG recording of a patient with multiple artifacts in baseline signal, impeding an accurate analysis of QRS complexes. (C) Interpretable ECG recording.

Both tests showed an appropriate inter-methods agreement, as shown in Table 2. Analyzing feasibility of Nuubo system in sports field, we found that ECG signal was adequate and interpretable in 16 players (84.2%) both at rest and during the three phases of the exercise: warming up, YYIRT and recovery time, clearly differentiated in activity index graph (Fig. 1). All these individuals presented mild signal abnormalities affecting less than three consecutive QRS complexes that did not prevent a proper interpretation of ECG, inasmuch as nECG suite software analysis was able to differentiate adequate QRS complexes from those considered artifact.

**Table 2:** Comparative Results Between Yo-Yo Test With Nuubo’s Technology and Ergospirometry

	ICC (95% CI)	P value
Quantitative variables		
Baseline heart rate	0.95 (0.87 - 0.97)	< 0.001
Peak heart rate	0.61 (0.21 - 0.83)	0.003
VO_2_ max	0.84 (0.63 - 0.93)	< 0.001

	Kappa index	P value
Qualitative variables		
Adequate baseline ECG	1	< 0.001
Adequate peak ECG	0.45	0.018
Atrial PC	1	< 0.001
Ventricular PC	1	< 0.001
ST segment depression	1	< 0.001

ICC: intra-class correlation coefficient; CI: confidence interval; VO_2_: maximal oxygen uptake; PC: premature complex.

When analyzing cardiac abnormalities, no ventricular extrasystoles or other arrhythmias were detected during any tests. Nor significant ST depression was detected in any player during both methods.

**Figure 2:**
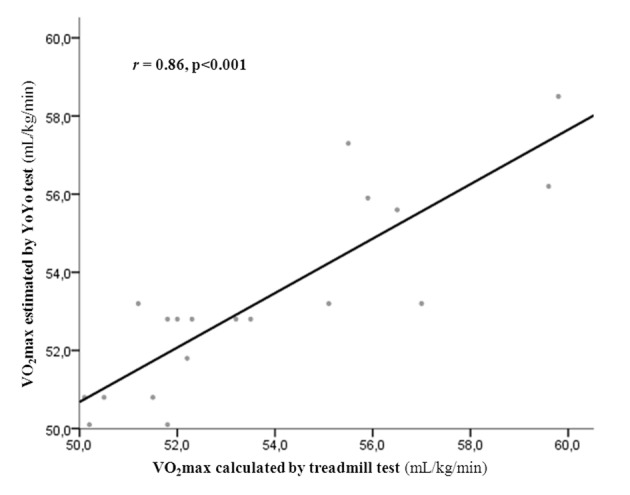
Relationship between treadmill ergospirometry and Yo-Yo intermittent recovery test level 1 in the calculation of maximum oxygen consumption (VO_2_ max).

In order to assess the usefulness of Nuubo system during YYIRT to determine physical performance variables compared with ergospirometry, we found a good correlation in calculation of VO_2_ max (VO_2_ max 53.3 ± 2.4 vs. 53.7 ± 3.0 mL/kg/min for Yo-Yo test and ergometry respectively; intra-class correlation coefficient 0.84 (0.63 - 0.93), P < 0.001) (Fig. 2). Maximal heart rate and maximal lactate reached by players are similar in both tests (comparisons using *t*-test: 187.7 ± 3.8 vs. 190.1 ± 5.5 beats per minute, P = 0.8; 10.4 ± 1.7 vs. 8.5 ± 3.3 mmol/L, P = 0.3; for Yo-Yo test and ergometry respectively). In addition, ergospirometry allowed the calculation of aerobic and anaerobic thresholds adequately (28.5 ± 6.1 and 38.5 ± 4.1 mL/kg/min respectively).

## Discussion

Screening of cardiac rhythm and conduction disturbances is universally supported to identify athletes with pre-existing conditions that place them at risk of catastrophic injury or sudden death. The pre-participation assessment as recommended by current guidelines involves a focused player and family medical history, a cardiac specific physical examination and resting 12-lead ECG [14].

Although other diagnostic tests are not initially required, the prognostic value of exercise testing in competitive athletes is undoubted. At present, although ergospirometry is widely used by elite teams because it allows direct estimation of performance variables such as VO_2_ max or aerobic/anaerobic thresholds, unfortunately it has the disadvantage of the need to travel to a hospital with consequent loss of training sessions. Furthermore, treadmill test is a continuous and progressive test that is poorly adapted to efforts of soccer players during trainings and matches. Thus, the implementation of medical tests is imperative in sports field that would allow continuous and reliable cardiac monitoring of soccer players since they represent an unsolved problem.

Accordingly, the use of electronic devices adapted to clothing has been recently evaluated in outpatients as those in cardiac rehabilitation [15]. Nonetheless, those have been poorly studied in sport as yet [16]. In this pilot study, Nuubo demonstrates for the first time its applicability in monitoring soccer players during a stress test in field, and seems to be a suitable system to reliably detect arrhythmias and calculate HRV parameters. Further studies will therefore be needed to implement this technology in the context described.

The main limitation of the study is the small sample during a single test. However, since it is a novel technology, this work could serve to spread this system in screening heart disease and assess the physical performance of soccer teams.

### Conclusions

The use of Nuubo’s technology allows an accurate single-lead electrocardiographic recording and estimation of reliable performance variables during exercise testing in field, and provides a new perspective to cardiac remote monitoring in team sports as soccer.
